# Erythropoietin in traumatic brain injury: study protocol for a randomised controlled trial

**DOI:** 10.1186/s13063-014-0528-6

**Published:** 2015-02-08

**Authors:** Alistair Nichol, Craig French, Lorraine Little, Jeffrey Presneill, D James Cooper, Samir Haddad, Jacques Duranteau, Olivier Huet, Markus Skrifvars, Yaseen Arabi, Rinaldo Bellomo, On behalf of the EPO-TBI Investigators and the Australian and New Zealand Intensive Care Society Clinical Trials Group

**Affiliations:** Australian and New Zealand Intensive Care Research Centre, School of Public Health and Preventive Medicine, Monash University, 99 Commercial Road, Melbourne, 3004 Australia; Department of Intensive Care Medicine, The Alfred, Commercial Road, Melbourne, 3004 Australia; Department of Anaesthesia and Intensive Care Medicine, St Vincent’s University Hospital, Elm Park, Dublin 4, Ireland; School of Medicine and Medical Sciences, University College Dublin, Elm Park, Dublin 4, Ireland; Department of Intensive Care, Western Health, Gordon Street, Footscray, 3011 Australia; Department of Medicine North West Academic Centre, The University of Melbourne, Grattan Street, Parkville, 3052 Australia; Department of Intensive Care, Mater Health Services, Raymond Terrace, South Brisbane, 4101 Australia; Department of Intensive Care, King Abdulaziz Medical City, PO Box 22490, Riyadh, 11426 Kingdom of Saudi Arabia; Service d’Anesthésie-Réanimation, Hôpitaux universitaires Paris Sud, Assistance Publique des Hôpitaux de Paris, Hôpital de Bicêtre, 78, rue du Général Leclerc, 94275 Le Kremlin Bicêtre, France; Department of Anesthesiology and Intensive Care Medicine, Centre Hospitalier Universitaire La Cavale Blanche Université de Bretagne Ouest, 29609 Brest Cedex, France; Department of Anaesthesiology and Intensive Care Medicine, Helsinki University Hospital, PO Box 266, FIN-00029 Helsinki, Finland; Intensive Care Department, College of Medicine King Saud Bin Abdulaziz University for Health Sciences and King Abdullah International Medical Research Center, PO Box 22490, Riyadh, 11426 Kingdom of Saudi Arabia; Department of Intensive Care, Austin Health, Studley Road, Heidelberg, 3084 Australia

**Keywords:** Traumatic brain injury, Erythropoietin, Outcome, Critical care, Randomised controlled trials

## Abstract

**Background:**

Traumatic brain injury is a leading cause of death and disability worldwide. Laboratory and clinical studies demonstrate a possible beneficial effect of erythropoietin in improving outcomes in the traumatic brain injury cohort. However, there are concerns regarding the association of erythropoietin and thrombosis in the critically ill. A large-scale, multi-centre, blinded, parallel-group, placebo-controlled, randomised trial is currently underway to address this hypothesis.

**Methods/design:**

The erythropoietin in traumatic brain injury trial is a stratified prospective, multi-centre, randomised, blinded, parallel-group, placebo-controlled phase III trial. It aims to determine whether the administration of erythropoietin compared to placebo improves neurological outcome in patients with moderate or severe traumatic brain injury at six months after injury. The trial is designed to recruit 606 patients between 15 and 65 years of age with severe (Glasgow Coma Score: 3 to 8) or moderate (Glasgow Coma Score: 9 to 12) traumatic brain injury in Australia, New Zealand, Kingdom of Saudi Arabia, France, Finland, Germany and Ireland.

Trial patients will receive either subcutaneous erythropoietin or placebo within 24 hours of injury, and weekly thereafter for up to three doses during the intensive care unit admission. The primary outcome will be the combined proportion of unfavourable neurological outcomes at six months: severe disability or death. Secondary outcomes will include the rate of proximal deep venous thrombosis detected by compression Doppler ultrasound, six-month mortality, the proportion of patients with composite vascular events (deep venous thrombosis, pulmonary embolism, myocardial infarction, cardiac arrest and cerebrovascular events) at six months and quality of life with health economic evaluations.

**Discussion:**

When completed, the trial aims to provide evidence on the efficacy and safety of erythropoietin in traumatic brain injury patients, and to provide clear guidance for clinicians in their management of this devastating condition.

**Trial registration:**

Australian New Zealand Clinical Trials registry: ACTRN12609000827235 (registered on 22 September 2009).

Clinicaltrials.gov: NCT00987454 (registered on 29 September 2009). European Drug Regulatory Authorities Clinical Trials: 2011-005235-22 (registered on 18 January 2012).

**Electronic supplementary material:**

The online version of this article (doi:10.1186/s13063-014-0528-6) contains supplementary material, which is available to authorized users.

## Background

Traumatic brain injury (TBI) is a devastating condition which affects close to 1,000 people each year in Australia, causes extensive long-term disability and suffering, which subsequently results in approximately 1 billion Australian dollars in lifetimes costs per year [1]. Disability follows from primary and secondary brain injury. Attenuation of secondary brain injury (decreasing the additional injury due to the inflammatory, excitotoxic and apoptotic response to trauma) is possible [2]. Erythropoietin (EPO) is a glycoprotein hormone with pleiotropic cytokine-like effects [3]. EPO has effects, independent of those on erythropoiesis, which are relevant to patients who have had a TBI. They include anti-apoptotic activity and protective neurological effects in the presence of hypoxia and ischaemia [2-4]. A neuroprotective effect of EPO has been demonstrated in animal models of TBI [5,6]. A large, multi-centre, randomised controlled trial (RCT) demonstrated that EPO improved survival in a retrospectively identified trauma cohort [7]. A subsequent large RCT by the same group found that while EPO did not reduce transfusion rates, in a prospectively identified trauma cohort (including TBI) EPO significantly decreased 29-day mortality compared to a placebo (3.5 versus 6.6%) [8]. Additional observational studies and one very small RCT support the hypothesis that EPO may improve neurological outcomes after TBI [9,10]. However, many concerns have been raised about the ability of EPO to increase the risk of venous thromboembolism (VTE) [8,11,12]. The United States Food and Drug Administration recently added a black box warning regarding this risk on EPO preparations. However, it is worth noting that the largest trial in the critically ill to date demonstrates a clear survival advantage in traumatically injured patients who are generally most vulnerable to VTE complications.

The question of whether the administration of EPO benefits patients with TBI remains unanswered. To address this evidence gap we are undertaking a large multi-centre, blinded, parallel-group, placebo-controlled randomised trial comparing the administration of EPO with a placebo. This trial will be sufficiently powered to detect clinically relevant differences in neurological outcomes measured by the eight-level Extended Glasgow Outcome Scale (GOSE) [13] at six months following injury.

## Methods/design

### Trial design and outcomes

The erythropoietin in traumatic brain injury (EPO-TBI) trial is a stratified prospective, multi-centre, randomised, blinded, parallel-group, placebo-controlled phase III trial. It aims to determine whether the administration of EPO compared to a placebo improves neurological outcome in patients with moderate or severe TBI at six months after injury. The trial is designed to recruit 606 patients between 15 and 65 years of age with severe (Glasgow Coma Score(GCS): 3 to 8) or moderate (GCS: 9 to 12) TBI in Australia, New Zealand, Kingdom of Saudi Arabia, France, Finland, Germany and Ireland.

### Participants

Patients aged between 15 and 65 years with non-penetrating moderate or severe TBI will be eligible for this trial. These criteria were designed to exclude patients with unsurvivable neurological injury, patients at a high risk of VTE and those who would be exposed to additional risk due to the trial drug.

### Inclusion criteria

Patients with non-penetrating moderate (Glasgow Coma Score (GCS) 9–12) or severe (GCS 3–8) traumatic brain injury admitted to an ICU who:

1. Are ≥15 to ≤65 years of age^a^
2. Are <24 hours since primary traumatic injury
3. Are expected to stay ≥48 hours
4. Have a haemoglobin not exceeding the upper limit of the applicable normal reference range in clinical use at the treating institution^b^
5. Have written informed consent from legal surrogate

### Exclusion criteria

Patients are excluded from the study if any of the following criteria apply^c^:

1. GCS = 3 and fixed dilated pupils
2. History of deep vein thrombosis, pulmonary embolism or other thromboembolic event
3. A chronic hypercoagulable disorder, including known malignancy
4. Treatment with erythropoietin in the last 30 days
5. First dose of study drug unable to be given within 24 hours of primary injury
6. Pregnancy or lactation or 3 months post-partum
7. Uncontrolled hypertension (systolic blood pressure >200 mmHg or diastolic blood pressure >110 mmHg)
8. Acute myocardial infarct within the past 12 months
9. Past history of epilepsy with seizures in past 3 months
10. Expected to die imminently (<24 hours)
11. Inability to perform lower limb ultrasounds
12. Known sensitivity to mammalian cell-derived products
13. Hypersensitivity to the active substance or to any of the additives
14. Pure red cell aplasia
15. End-stage renal failure (receives chronic dialysis)
16. Severe pre-existing physical or mental disability or severe co-morbidity that may interfere with the assessment of outcome
17. Spinal cord injury
18. Treatment with any investigational drug within 30 days before enrolment
19. The treating physician believes it is not in the best interests of the patient to be randomised to this trial

### Outcomes measures

The primary outcome of this trial is the patients’ neurological status at six months, summarized as a binary midpoint reduction of their eight-level Extended Glasgow Outcome Scale (GOSE) score, defined as favourable (GOSE score: 5 to 8; moderate disability and good recovery) or unfavourable (GOSE score: 1 to 4; death and severe disability).

Secondary outcomes include:

1. Neurological status at six months summarized by the eight-level GOSE
2. Quality of life assessment using the Short Form 12 (SF-12) [14] and EuroQoL-5D (EQ-5D) [15] at six months
3. Mortality at six months
4. Proximal deep venous thrombosis (DVT) detected by ultrasound
5. Occurrence of a thrombotic vascular event (including DVT, pulmonary embolism, myocardial infarction, cardiac arrest and cerebrovascular events) at six months. The definitions for these events are previously reported [16]
6. Resource use and costs at six months
7. Incremental cost effectiveness

### Trial interventions

The intervention to be examined in this trial is the subcutaneous administration of erythropoietin 40,000 International Units (IU) compared to a placebo (0.9% saline).

**Figure 1:**
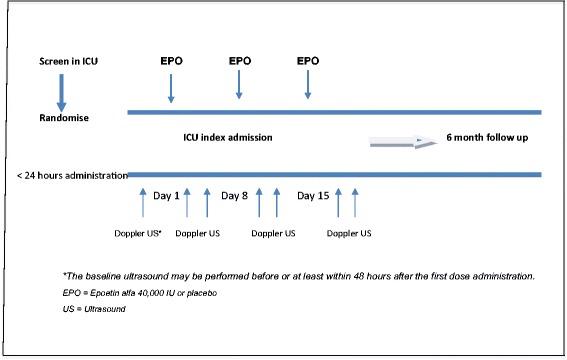
**Summary of trial treatment and follow up schedule.** The baseline ultrasound may be performed before or at least within 48 hours after the first dose administration. EPO, Erythropoietin (Epoetin alfa) 40,000 IU or normal saline placebo; US, Ultrasound.

A summary of the trial interventions and follow up schedule is provided in Figure 1.

### Investigational product

The trial drug is Epoetin alfa 40,000 IU in a pre-filled syringe, manufactured by Janssen-Cilag Pty Ltd, or the placebo sodium chloride 0.9. The trial drug will be administered by subcutaneous injection.

For patients randomised to the treatment arm, an unblinded nurse will administer the full contents of the Epoetin alfa 40,000 IU pre-filled syringe to the patient. For patients randomised to the placebo arm, an unblinded nurse will administer 1 mL from a sodium chloride 0.9% 10 mL ampoule.

The first dose will be given within 24 hours of the estimated time of TBI, and then weekly for up to two more doses (on trial days eight and 15). The trial drug will only be administered to patients during the ICU admission. If the patient is discharged to the general ward (before trial day eight or before day 15) they will not receive any further doses of trial drug. Patients readmitted to ICU during the same hospital stay will not receive any further doses of the trial drug.

### Variable haemoglobin threshold for dosing to reduce the risk of thromboembolic complications

Previous trials examining the long-term use of EPO in renal failure [12] and cancer [11] patients targeting haemoglobin (Hb) concentrations above 120 g/L (12 g/dL) have demonstrated an increased risk of thromboembolic complications. Observational studies also demonstrate that the Hb concentration in critically ill patients falls rapidly in the first 72 hours of admission [17,18]. A dosing structure based on Hb concentration was developed to address the known risks of thromboembolic complications associated with EPO use: the first dose of trial drug is administered only if the haemoglobin (Hb) concentration is less than the upper limit of the applicable normal reference range in clinical use at the treating institution; the second and third doses are administered only if the Hb concentration is <120 g/L (12 g/dL).

The second (trial day eight) and third (trial day 15) doses may be given the day before or after dosing day, so the trial drug is prepared in pharmacy business hours. The weekly dose will not be administered if the patient’s pre-medication Hb is ≥120 g/L (12 g/dL); the trial drug will be temporarily withheld for that dose. The patient will be assessed on the next scheduled dosing day. If their Hb is <120 g/L (12 g/dL), the dose may be given if the patient is still in the ICU and has not met the permanent withholding criteria.

At Johannes Gutenberg-Universtität, Mainz Germany an additional temporary trial drug withholding criterion applies. The weekly dose will not be administered if the patient has a refractory high blood pressure; the trial drug will be withheld for that dose, and the patient will be assessed on the next scheduled dosing day. No further doses will be administered if the patient has met any permanent withholding criteria.

Permanent withholding criteria:

1. Development of proximal deep venous thrombosis
2. Development of pulmonary embolism
3. Any other thrombotic event
4. Acute myocardial infarction
5. Cardiac arrest or ventricular fibrillation
6. Cerebrovascular accident
7. Any serious adverse event or protocol deviation where, in the attending physician’s opinion, the patient should not receive any further doses of the trial drug
8. Consent has been withdrawn or consent to continue has not been granted

### Management of traumatic brain injury

The ICU medical team will have full independent control of patient management, however the trial management committee request that standardised TBI clinical practice follows Brain Trauma Foundation [19] guidelines.

### Compression Doppler ultrasound

Critically ill patients are at risk of VTE complications. Previous studies, which did not specifically screen for VTE, have demonstrated an association of EPO with increased rates of VTE. The true risk is unknown in the critically ill as clinical examination has severe limitations, and many clinically undetected VTE complications are revealed when prospective screening is performed. The risk of VTE in the traumatically injured patients is potentially further elevated, as concerns regarding intracerebral haemorrhage frequently discourage clinicians giving pharmaceutical agents (low molecular weight heparins) to mitigate this risk. Therefore, to address this potential and largely unknown risk of EPO in the traumatically injured patients, we designed a prospective VTE screening algorithm. Bilateral compression Doppler ultrasound of the lower extremities will be performed to monitor for proximal DVT at baseline (before the first dose if possible, or at least within 48 hours after the first dose administration) then twice weekly after each dose of the trial drug for three weeks or up to ICU discharge, whichever occurs first. The procedure for the ultrasound was standardised, with evaluation of the following veins: common femoral, proximal femoral, mid femoral, distal femoral, popliteal and trifurcation.

Up to six scheduled twice weekly ultrasounds will be performed if the patient is an inpatient in the ICU for longer than three weeks. No further scheduled twice weekly ultrasounds are required beyond trial day 21.

If the patient meets a temporary or permanent withholding criteria and remains in the ICU, the twice weekly compression Doppler ultrasounds are performed up to trial day 21 or ICU discharge, whichever occurs first.

Bilateral compression Doppler ultrasounds will be performed on the general ward if the patient has been transferred after a dose of the trial drug and before a scheduled ultrasound has been performed. This will ensure at least one bilateral compression Doppler ultrasound will be performed after each dose of the trial drug.

The site treating clinicians outside the ICU (known variously by names such as Parent Unit, Admitting Unit or Attending Medical Service) are required to notify the site researchers of any occurrences of VTE as soon as it is diagnosed.

Additional bilateral compression Doppler ultrasounds will be performed on suspicion of DVT or pulmonary embolism. The treating clinician may therefore take additional measures to reduce the risk of complications from any identified thrombosis. We anticipate that this algorithm will mitigate any additional risk that could be posed by the use of EPO in this cohort.

### Randomisation and allocation concealment

Concealed randomisation will be performed via a web-based system that includes block randomisation at each site. Treatment allocation will be stratified by site and also by the severity of TBI at randomisation (moderate (GCS: 9 to 12) or severe (GCS: 3 to 8).

Bias will be minimised by concealed random allocation by the use of a robust primary outcome minimally susceptible to ascertainment bias, and by blinded assessment of the primary outcome by a trained outcome assessor at the coordinating centre (Australia and New Zealand) or by trained, blinded outcome assessors in each country in Europe and the Kingdom of Saudi Arabia.

### Blinding plan

Site personnel will be blinded to treatment allocation. For the efficient conduct of the trial the site pharmacists, site unblinded dosing nurses and the pharmacists at the central pharmacy in France (Clinical Trial Department of the Pharmaceutical Establishment of Assistance Publique-Hôpitaux de Paris) will be unblinded to treatment assignment.

Staff at the coordinating centre will be blinded to treatment allocation, except for an unblinded project officer (Appendix 1) and designee, the analyst programmers responsible for the web-based data management system and a nominated statistician who will supervise data extraction from the database for interim and final analyses. The French management team will be blinded to treatment assignment.

**Figure 2:**
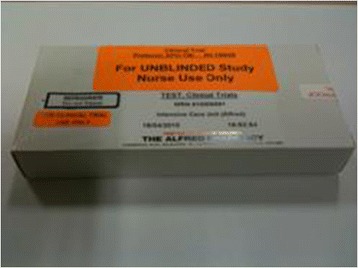
**Blinded tamperproof sealed box.**

Following patient randomisation by the site investigator or designee, an unblinded trial pharmacist will dispense the trial drug in a tamperproof sealed opaque box (Figure 2) to blind the treatment assignment. The box may only be opened by the site’s designated unblinded trial dosing nurse. For patients who receive the active treatment the sealed box will consist of one Epoetin alfa 40,000 IU pre-filled syringe labelled with direction for use ‘Inject 1 ml subcutaneously over at least 1 minute’. For patients who receive the placebo the sealed box will consist of one sodium chloride 0.9% 10 mL ampoule labelled with direction for use ‘Inject 1 ml subcutaneously over at least 1 minute’.

For safety reasons, unblinded dosing nurses will be allocated in each research site to administer EPO or placebo doses discreetly, with a screen around the patient bed area. The trial drug or placebo dose may be checked with a second unblinded nurse if required to comply with local hospital regulations. After dose administration the unblinded dosing nurse will discard the used trial drug in such a manner as to maintain the blind. These unblinded dosing nurses will have access to the unblinded trial pharmacist. Unblinded dosing nurses will not be involved in the care of a trial patient and may not discuss trial drug treatment with research staff or other members of the ICU or hospital staff.

### Data collection and management

All data will be collected by trained staff at each trial site using a paper source document developed by the coordinating centre. Data will then be entered into a web database designed by the trial project manager in collaboration with the Monash University Monash University Clinical Informatics and Data Management Unit. Data queries will be automatically generated via the electronic data collection database, and at monitoring by the trial project manager or the clinical research associate (in France).

Randomised patients will be followed up to death or six months post-randomisation (whichever occurs first). Data collection will be restricted primarily to those variables necessary to define clinical patient characteristics including: baseline demographics, primary diagnoses, physiological parameters, Acute Physiology and Chronic Health Evaluation II (APACHE II), Injury Severity Score (ISS), Abbreviated Injury Severity Score (AIS) and Computed Tomography Brain Marshall Score [20], diagnostic interventions, therapeutic interventions, and documentation of deaths and other serious adverse events (SAE).

To prepare for the six-month follow-up assessment, patients and/or their legal surrogate will be asked to provide three possible points of contact (home and close family contact details) to the research staff prior to hospital discharge. Full protocol data will be collected in all patients including those excluded at any stage. Patients who are alive at six months after randomisation (or their carer or proxy if more appropriate) will be interviewed by a trained outcome assessor from Monash University for patients in Australia and New Zealand, or trained outcome assessors in each country in Europe and the Kingdom of Saudi Arabia. The regional assessors will use a standardized structured telephone questionnaire [21] to measure the eight-level GOSE [13]. Neurological outcomes will then be defined as favourable (GOSE score: 5 to 8; moderate disability and good recovery) or unfavourable (GOSE score: 1 to 4; death and severe disability). Patients subsequently withdrawn for any reason (DVT, pulmonary embolism, myocardial infarction, cerebrovascular accident, cardiac arrest, or an SAE) or who did not receive the trial drug will be followed up on, according to the study follow-up schedule, and analysed according to the modified intention-to-treat principle [22,23].

Trained outcome assessors (Appendix 2) will collect data for surviving patients at six months for quality of life assessment using the EQ-5D [15] questionnaire and the SF-12 [14] questionnaire. If the patient is not well enough or not available to complete the questionnaires, the SF-12 questionnaire will not be completed [24,25] and quality of life will not be assessed for that patient.

Data will be collected from surviving patients at six months for cost effectiveness analysis, with utilities calculated using the EQ-5D [15] results, and costs will be calculated based on ICU, acute and post-acute care resource use. If the patient is not well enough or not available to complete the EQ-5D the patient’s proxy will be asked to complete it for the patient.

The outcome assessments will be monitored by an experienced and trained outcome assessor at Monash University.

### Ethical issues

This is a trial conducted in patients who are unconscious and unable to consent to participation, therefore the patient’s legal surrogate will be approached to provide consent for the patient. Patients who recover sufficient cognition to understand the explanation of the trial will additionally be asked to consent to continue in the trial if this is required under the ethics committee approval conditions.

In France patients may be enrolled under an Emergency clause. Informed consent was obtained from each patient’s legal surrogate for participation in the trial.

Approval for this protocol has been obtained from appropriate regulatory authorities, and from participating hospitals’ human research ethics committees. The list of responsible ethics committees is provided as an Additional file 1.

### Sample size and power

The estimated rate of unfavourable neurological outcome (death and severe disability) in Australian and New Zealand patients with moderate and severe TBI [26,27] is approximately 50%.

A trial of 574 patients will have a 90% power at an alpha of 0.05 to detect a 14% absolute risk reduction (50 versus 36%) and 80% power to detect a 12% (50 versus 38%) absolute risk reduction in unfavourable neurological outcome. A trial of this size was also estimated to have 80% power to detect a 9% absolute risk increase in proximal lower limb DVT from an assumed baseline proportion of 18% (50% increase in relative risk) at a one-sided alpha of 0.05. Allowing for a 5% withdrawal and loss to follow-up rate, we will recruit 606 patients to prevent any loss of power and to conduct an adequately powered modified intention-to-treat analysis [22,23].

### Statistical analysis

Independent senior statisticians at Monash University Department of Epidemiology and Preventive Medicine will perform data analyses following a detailed statistical analysis plan, which will be published separately in the journal *Trials*.

A modified intention-to-treat analysis will be performed based on all randomly assigned patients, except those withdrawing consent for use of all trial data, those not fulfilling inclusion criteria and those who never receive the intervention [22,23]. Baseline variables will be summarised using descriptive statistics. The trial primary outcome will be compared between treatments with an unadjusted risk ratio and 95% confidence interval. Sensitivity analyses will be performed using logistic regression adjusting for stratification factors, pre-specified prognostic factors and any other baseline covariates exhibiting substantial imbalance between randomisation arms. Furthermore, a proportional odds cumulative logit model [28,29], adjusting for relevant covariates, will be applied to the eight-level vector of the six-month GOSE score.

Other secondary analyses, including assessment of outcomes according to actual treatment received, quality of life assessment, mortality at hospital discharge and six months and incidence of adverse events (AE), will be compared between treatment groups using unadjusted and adjusted logistic regression and log-binomial regression. Pre-specified subgroup analyses will be obtained using interaction terms in logistic regression models. Adjusted effect estimates of the EPO intervention, derived from logistic and proportional odds ordinal logistic models, will be reported as adjusted risk ratios averaged over the remaining covariates, as recently recommended [30-32]. Time-to-event analyses will be undertaken using Kaplan-Meier curves, as well as unadjusted and adjusted Cox proportional hazards regression models.

### Data and safety monitoring

An independent Data Safety and Monitoring Committee (DSMC) comprising of experts in clinical trials, biostatistics and intensive care, will monitor SAEs throughout the trial, and pre-defined outcomes at designated interim analyses.

Given the potential for EPO to increase the risk of VTE, there are two planned interim safety analyses scheduled by the DSMC at six months, following 33% (n = 202) and 66% (n = 404) patient recruitment. The Haybittle-Peto criterion (|Z_k_| > =3) for early stopping were applied at these first and second analyses. The final analyses at full recruitment will be little affected by these interim analyses (final critical value |Z_3_| > =1.975 rather than 1.960) [33].

Consistent with other studies in critically ill patients, AEs already defined and reported as study outcomes (apart from death) will not be reported a second time as SAEs [34].

## Discussion

TBI is a common and devastating condition with few proven specific therapies available. The administration of EPO has the potential to reduce neurological damage and improve outcome, and is supported by a scientific rationale and laboratory data. The EPO-TBI design aims to maximise the ability to detect a beneficial effect, if one exists, between EPO and improved neurological function after TBI. Furthermore, our design features also aim to minimise the risk of VTE in this population, and to develop a prospective screening plan which will readily identify VTE events if they occur, allowing clinicians to provide appropriate treatment rapidly. EPO-TBI aims to provide definitive guidance for clinicians regarding the true efficacy and safety of EPO in the management of TBI.

### Trial status

The trial commenced in May 2010 at The Alfred Hospital, Melbourne Australia. Two interim analyses were conducted with approval by the DSMC to continue the trial without alteration to the protocol. The target recruitment of 606 patients was achieved on 1 November 2014, making final six-month outcomes available by May 2015.

## Endnotes

^a^Six sites have a minimum age of 15 years, 13 sites have a minimum age of 16 years and 10 sites have a minimum age of 18 years.

^b^<140 g/L at Johannes Gutenberg-Universtität, Mainz Germany, <148 g/L for males and <135 g/L for females at Royal Adelaide Hospital, Adelaide Australia.

^c^Additional exclusion criteria at Johannes Gutenberg-Universtität, Mainz Germany. Uncontrolled hypertension parameters were more stringent (systolic blood pressure of >160 mm Hg or diastolic blood pressure of >90 mm Hg), morbid obesity, coronary artery disease, peripheral arterial occlusive disease, vascular disease of the carotid arteries, cerebrovascular disorders, recent stroke, contraindications against prophylaxis of DVT or an increased risk for DVT (for example, with additional trauma and /or operations, severe varicose veins, severe smokers, intake of oral contraceptives, infections and inflammation).

## Appendix 1: EPO-TBI unblinded project officer

Belinda Howe, Australian and New Zealand Intensive Care Research Centre, Monash University, Melbourne, Australia.

## Appendix 2: EPO-TBI outcome assessors

Heather Waddy, Australian and New Zealand Intensive Care Research Centre, Monash University, Melbourne, Australia.

Marwan Al Kishi, Department of Medicine, King Abdulaziz Medical City, Riyadh, Kingdom of Saudi Arabia.

Sarah Kambire, Unité de Recherche Clinique Lariboisière-Saint Louis, Assistance Publique-Hôpitaux de Paris, France.

Serge Camelo, Unité de Recherche Clinique Lariboisière-Saint Louis, Assistance Publique-Hôpitaux de Paris, France.

Markus Skrifvars, Intensive Care Unit, Department of Anaesthesiology and Intensive Care Medicine, Helsinki University Hospital, Helsinki, Finland.

Stepani Bendel, Intensive Care Unit, Division of Anaesthesiology and Intensive Care Medicine, Kuopio University Hospital, Kuopio, Finland.

Carole Schilling, Royal College of Surgeons in Ireland, Education and Research Centre, Beaumont Hospital, Dublin, Ireland.

Thomas Kerz, Department of Neurosurgery, Intensive Care Therapy Unit, Universitätsmedizin, Mainz, Germany.

Lynnette Murray, Australian and New Zealand Intensive Care Research Centre, Monash University, Melbourne, Australia.

## Appendix 3: EPO-TBI management committee

Rinaldo Bellomo, Department of Intensive Care, Austin Health, Melbourne, Australia.

Alistair Nichol, Department of Anaesthesia and Intensive Care Medicine, St Vincent’s University Hospital, Dublin, Ireland.

Craig French, Department of Intensive Care, Western Health, Melbourne, Australia.

D James Cooper, Department of Intensive Care Medicine, The Alfred, Melbourne, Australia.

Olivier Huet, Intensive Care Unit, Department of Anesthesiology and Intensive Care Medicine, Centre Hospitalier Universitaire La Cavale Blanche, Brest, France.

Lorraine Little, Australian and New Zealand Intensive Care Research Centre, Monash University, Melbourne, Australia.

Anne Mak, Pharmacy Department, The Alfred, Melbourne, Australia.

Ville Pettilä, Intensive care Units, Division of Anaesthesiology and Intensive Care Medicine, Helsinki University Central Hospital, Helsinki, Finland.

Jeffrey Presneill, Department of Intensive Care, Mater Health Services, Brisbane, Australia.

Shirley Vallance, Department of Intensive Care Medicine, The Alfred, Melbourne, Australia.

Dinesh Varma, Department of Radiology, The Alfred, Melbourne, Australia.

Judy Wills, Department of Radiology, The Alfred, Melbourne, Australia.

## Appendix 4: EPO-TBI sites, principal investigator and research coordinator/s

Auckland City Hospital, New Zealand: Colin McArthur, Yan Chen, Lynette Newby.

Beaumont Hospital, Ireland: Criona Walshe, James O’Rourke, Carole Schilling.

Canberra Hospital, Australia: Imogen Mitchell, Frank Van Haren, Helen Rodgers. Marta Kot

Christchurch Hospital, New Zealand: Seton Henderson, Jan Mehrtens, Sascha Noble.

Dunedin Hospital, New Zealand: Matthew Bailey, Robyn Hutchinson, Dawn France.

Gold Coast University Hospital, Australia: Brent Richards, Mandy Tallott.

Helsinki University Central Hospital, Finland: Markus Skrifvars, Heikki Vartiala, Marianne Eliasson.

Hôpital Caremeau, France: Jean Yves Lefrant, Laurent Muller, Claire Roger, Christian Bengler, Pierre Barbaste.

Hôpital Charles Nicolle, France: Benoit Veber, Marie Gilles-Baray, Pierre-Gildas Guitard, Helene Braud.

Hôpital de Bicêtre, France: Jacques Duranteau, Anatole Harrois, Samy Figueiredo, Sophie Hamada.

Hôpital Lariboisière, France: Didier Payen, Anne Claire Lukaszewicz, Charles Damoisel, Sarah Kambire.

Hôpital Michallon, France: Jean François Payen, Pauline Manhes, Gilles Franconey, Perrine Boucheix.

Johannes Gutenberg-Universtität, Germany: Thomas Kerz.

John Hunter Hospital, Australia: Peter Harrigan, Miranda Hardie.

King Abdulaziz Medical City, Kingdom of Saudi Arabia: Samir Haddad, Yaseen Arabi, Marwan Al Kishi, Ahmad Deeb, Shmeylan Al Harbi, Lolowa Al-Swaidan ,Turki Al Moammar, Juliet Lingling, Shella Caliwag, Hanie Richi.

Kuopio University Hospital, Finland: Stepani Bendel, Sari Rahikainen, Mikko Myllymaki.

Liverpool Hospital, Australia: Victor Tam, Sharon Micallef.

Nepean Hospital, Australia: Louise Cole, Leonie Weisbrodt.

Royal Adelaide Hospital, Australia: Richard Strickland, Justine Rivett, Sonya Kloeden, Stephanie O’Connor.

Royal Hobart Hospital, Australia: David Cooper, Richard McAllister.

Royal Melbourne Hospital, Australia: Nerina Harley, Deborah Barge, Elizabeth Moore, Andrea Jordan.

Royal North Shore Hospital, Australia: Simon Finfer, Elizabeth Yarad, Simon Bird, Anne O’Connor.

Royal Perth Hospital, Australia: Geoffrey Dobb, Jenny Chamberlain, Michelle Barr, Elizabeth Jenkinson.

Royal Prince Alfred Hospital, Australia: David Gattas, Heidi Buhr, Debra Hutch, Megan Keir.

St Vincent’s Hospital Sydney, Australia: Priya Nair, Claire Reynolds, Serena Knowles.

The Alfred, Australia: D. James Cooper, Jasmin Board, Shirley Vallance, Phoebe McCracken.

The Townsville Hospital, Australia: Geoffrey Gordon, Stephen Reeves.

Wellington Regional Hospital, New Zealand: Richard Dinsdale, Lynn Andrews, Dianne Mackle, Sally Hurford.

Westmead Hospital, Australia: Vineet Nayyar, Christina Whitehead, Jing Kong.

## Appendix 5: EPO-TBI French management team

Jacques Duranteau, National Principal Investigator, Service d’Anesthésie-Réanimation, Hôpitaux universitaires Paris Sud, Assistance Publique des Hôpitaux de Paris, Hôpital de Bicêtre, Le Kremlin-Bicêtre, France.

Eric Vicaut, Unité de Recherche Clinique Lariboisière-Saint Louis, Assistance Publique-Hôpitaux de Paris, France.

Philippe Gallula, Pole Promotion International, Assistance Publique-Hôpitaux de Paris, France.

Vidhya Raghavan, Unité de Recherche Clinique Lariboisière-Saint Louis, Assistance Publique-Hôpitaux de Paris, France.

Amel Chamam, Unité de Recherche Clinique Lariboisière-Saint Louis, Assistance Publique-Hôpitaux de Paris, France.

Sarah Kambire, Unité de Recherche Clinique Lariboisière-Saint Louis, Assistance Publique-Hôpitaux de Paris, France.

## Additional file

Additional file 1:
**Project Title: Erythropoietin in traumatic brain injury.** List of participating centres and responsible ethics committees.

## Abbreviations

AE: Adverse event
AIS: Abbreviated injury severity score
ANZIC-RC: Australian and New Zealand Intensive Care Research Centre
APACHE II: Acute Physiology and Chronic Health Evaluation II
CI: Confidence interval
DSMC: Data and Safety Monitoring Committee
DVT: Deep venous thrombosis
EPO: Erythropoietin
EPO-TBI: Erythropoietin in Traumatic Brain Injury
EQ-5D: EuroQol five dimensions
GCS: Glasgow coma score
GOSE: Extended Glasgow outcome scale
Hb: Haemoglobin
ISS: Injury severity score
SF-12: Short Form-12
TBI: Traumatic brain injury
VTE: Venous thromboembolism
